# Supplementary material for: Evaluation of the Performance of Rapid Diagnostic Tests for Malaria Diagnosis and Mapping of Different Plasmodium Species in Mali
Source: Int J Environ Res Public Health. 2024 Feb 15;21(2):228. doi: 10.3390/ijerph21020228 (PMC10888130; doi:10.3390/ijerph21020228)
Supplement: Supplementary file 1 [file ijerph-21-00228-s001.zip › ijerph-2756714-supplementary.pdf]

Table S1. Detail of the total number and the prevalence of positive and negative Malaria Rapid Diagnostic Tests in each health center.

Area	Health district	RDT collection site	Result		Total
			Positive N (%)	Négative N (%)	
**Kayes**	Diéma	Torodo	6 (2.2)	272(97.8)	278
		Lakamané	0 (0.0)	89 (100)	89
		Lattakaf	0 (0.0)	47 (100)	47
		Débomassassi	0 (0.0)	48 (100)	48
		Koungo	3 (75.0)	1 (25.0)	4
		Lambidou	0 (0.0)	13 (100)	13
	Yélimané	kodié	12 (19.0)	51(81.0)	63
		Csréf	6 (4,1)	139 (95.9)	145
		Dogofry	0 (0.0)	23 (100.0)	23
		Bandiougoula	0 (0.0)	11 (100.0)	11
**sub total**			**27 (3.7)**	**694 (96.3)**	**721**
**Koulikoro**	Nara	Bagoini	0 (0.0)	50 (100.0)	50
		Mourdiah	0 (0.0)	68 (100.0)	68
		Kassakaré	4 (12.1)	29 (87.9)	33
		Alasso	1 (3.4)	28 (96.6)	29
		Tiapato	3 (14.3)	18 (85.7)	21
		Waourou	0 (0.0)	13 (100.0)	13
	Kangaba	Naréna	2 (1.6)	126 (98.4)	128
		Cscom Central	14 (28.0)	36 (72.0)	50
		Séléfougou	0 (0.0)	30 (100.0)	30
**sub total**			**24 (5.7)**	**398 (94.3)**	**422**
**Sikasso**	Kadiolo	cscom central	110 (59.5)	75 (40.5)	185
		Zégoua	28 (38.89)	44 (61.1)	72
**sub total**			**138 (53.7)**	**119 (46.3)**	**257**
**Segou**	Barouéli	Cscom Central	5 (41.7)	7 (58.3)	12
		Dioforogo	13 (65.0)	7 (35.0)	20

		Tamani	7 (35.0)	13 (65.0)	20
		NGara	16 (80.0)	4 (20.0)	20
		Tigui	20 (100.0)	0 (0.0)	20
		bananido	7 (50.0)	7 (50.0)	14
		N'Gossola	5 (31.3)	11(68.75)	16
		Nianzana	20 (66.7)	10 (33.3)	30
		yerebougou	16 (80.0)	4 (20.0)	20
		Csréf	8 (40.0)	12 (60.0)	20
		Ndjila	10 (50.0)	10 (50.0)	20
**sub total**			**127 (59.9)**	**85 (40.1)**	**212**
**Mopti**	Mopti	Soufouroulaye	79 (34.3)	151 (65.7)	230
		Fatoma	0 (0.0)	35 (100.0)	35
		Sévaré II	136 (91.9)	12 (8.1)	148
**sub total**			**215 (52.1)**	**198 (47.9)**	**413**
**Tombouctou**		Gourma Rharous	400 (66.9)	198 (33.1)	598
**Gao**	Gao	Csref d'Ansongo	30 (13.4)	194 (86.6)	224
**Menaka**	Ménaka	Menaka	21 (36.8)	36 (63.2)	57
**Kidal**	Kidal	Cscom d'Aliou	0 (0.0)	54 (100.0)	54
		CSRéf de Kidal	20 (10.3)	120 (85.7)	140
**sub total**			**20 (10.3)**	**174 (89.7)**	**194**
Total			**1002 (32.3)**	**2096 (67.7)**	**3098**

3098 RDT cassettes were collected at the various study sites, of which 67.7 were malaria-negatives.
